# Cytokine *TGF-β1, TNF-α*, *IFN-γ* and* IL‐6* Gene Polymorphisms and Localization of Premalignant Gastric Lesions in Immunohistochemically *H. pylori*-negative Patients

**DOI:** 10.7150/ijms.60517

**Published:** 2021-05-21

**Authors:** Anca Negovan, Mihaela Iancu, Florin Tripon, Andrei Crauciuc, Simona Mocan, Claudia Bănescu

**Affiliations:** 1 Department of Clinical Science-Internal Medicine, George Emil Palade University of Medicine, Pharmacy, Science, and Technology of Târgu Mureș, Mureș, Romania.; 2 Department of Medical Informatics and Biostatistics, Iuliu Hațieganu University of Medicine and Pharmacy, Cluj-Napoca, Cluj-Napoca, Romania.; 3 Genetics Laboratory, Center for Advanced Medical and Pharmaceutical Research, George Emil Palade University of Medicine, Pharmacy, Science and Technology of Târgu Mureș, Târgu Mureș, Romania.; 4 Department of Medical Genetics, George Emil Palade University of Medicine, Pharmacy, Science and Technology of Târgu Mureș, Târgu Mureș, Romania.; 5 Pathology Department, Emergency County Hospital Targu Mures, Mureș 540139, Romania.

**Keywords:** atrophic gastritis, intestinal metaplasia, cytokine polymorphism, *TGF*‐*β1*, * TNF‐α*, * IFN‐γ*, * IL*-6

## Abstract

**Background:** Cytokines and their gene variants are proven to play a role in pathogenic gastritis and carcinogenesis. The study assesses associations of the cytokine gene polymorphisms with extension of atrophic gastritis/intestinal metaplasia (AGIM) in patients without *Helicobacter pylori* infection on immunohistochemistry study.

**Methods:** 224 adult consecutive patients undergoing an upper digestive endoscopy were included and grouped according to localization of AGIM: 37 patients with antrum-limited AGIM, 21 corpus-limited AGIM, 15 extended-AGIM (antrum and corpus) and 151 patients had no AGIM. Medical records of the patients were checked and a structured direct interview was applied in order to collect clinical data, including digestive symptoms. In all cases, *IFN-γ* +874T>A, *TGF-β1* +869T>C, *TNF‐α*-308G>A and -238G>A, and *IL-6 -*174C>G polymorphisms were genotyped.

**Results:** The mean age was significantly higher in the AGIM group, while the comorbidies were similar among patients with different localization of lesions or in patients without AGIM. There were no significant differences in digestive symptoms, nor in the consumption of non-steroidal anti-inflammatory drugs or proton pump inhibitor with the different extensions of AGIM. There was a significant association between oral anticoagulant consumption and localization of AGIM (*P* = 0.042), frequency being higher among patients with corpus-limited AGIM than those with no AGIM (*P* = 0.007, adjusted *P* = 0.041).* TGF-β1* +869T>C was less frequent among patients with corpus-limited AGIM (*n*=7, 33.3%) and extended AGIM (*n*=5, 33.3%) than in antrum-limited AGIM (*n*=25, 67.6%). There were no other significant differences regarding variant and wild genotype frequencies of *IFN-γ* +874T>A (86.5%, 81.0%, 86.7%, p=0.814), *TNF‐α*-308G>A (35.1%, 28.6%, 53.3%, p=0.48) and *IL-6 -*174C>G (70.3%. 61.9%, 73.3% p=0.656) among patients with antrum-limited, corpus-limited or extended AGIM. *TGF-β1* +869T>C was associated with a decreased risk for corpus-affected AGIM (adjusted odds ratio: 0.42, 95% confidence interval: 0.19-0.93, *P* = 0.032). The dominant inheritance models no revealed significant association for *IFN-γ* +874T>A, *TNF‐α*-308G>A and *IL-6 -*174C>G gene polymorphism and the risk of localization of AGIM.

**Conclusion:** *TGF-β1* +869T>C gene polymorphism is associated with a decreased risk for corporeal localization of premalignant lesions, while *IFN-γ* +874T>A, *TNF-α*-308G>A and *IL-6 -*174C>G are not associated with the risk for AGIM in immunohistochemically *H. pylori* negative patients.

## Introduction

Gastric cancerogenesis is accepted today to be correlated with long-standing chronic inflammation in the stomach mucosa, most frequently due to *Helicobacter pylori* (*H. pylori*) infection 1. Both host and *H. pylori* genetic factors seem to play a crucial role in gastric inflammation and progression toward cancer 2. “Gastric cancer phenotype” described as corpus predominant gastritis, atrophic gastritis and/or intestinal metaplasia (AGIM), decreased acid secretion or *H. pylori* infection (present or previous) increases the risk for cancer 3. On the other hand, autoimmune atrophic gastritis, a condition diagnosed in the presence of corpus gastric atrophy with antrum sparing, can also lead to the development of adenocarcinoma or neuroendocrine neoplasia 4. Recent meta-analysis support that single nucleotide polymorphisms may be used for the assessment of genetic predisposition to gastric cancer, with ethnicity, environmental factor and cancer subtype contributing to inconsistency in the results 5. From among all molecular factors playing a role in progression from inflammation toward preneoplastic gastric lesions and cancer, recent research has focused on the possible influence of different type cytokine gene variants 6. Recently, it was shown that cytokine gene variants are associated with susceptibility to different types of malignancy in a Romanian population 7.

Experimental data support that the CD4+ T cell-derived interferon-gamma (IFN-γ) provides the key stimulus for the development of gastric premalignant lesions 8. The *IFN‐γ* +874T>A (rs2430561) polymorphism influencing IFN-γ expression has not been studied in relation to mucosal gastric lesion 9,10.

The transforming growth factor-β1 (*TGF-β1*) gene and its protein play an important role in modulating expression of multiple genes, being involved in inflammatory responses in gastric mucosa and cancer progression 11. Published meta-analysis did not confirm the association between *TGF-β1* +869T>C (rs1800470) and 915G>C (rs1800471) polymorphisms and the risk for gastric cancer 12, while another indicated that *TGF β1* -509C>T rather than +869T>C can increase the risk for gastric cancer 13. The functional *TGF-β1* +869T>C, rs1800470 polymorphisms have been identified to be associated with expression and the level of plasma TGF-β1 protein 11,14,15.

Tumor necrosis factor-alpha (TNF-α) is a potent immunomodulator and pro-inflammatory cytokine that inhibits gastric acid production, and it is upregulated in the gastric mucosa in response to *H. pylori* infection 16. Meta-analysis sustained the association between *TNF-*α -308G>A (rs1800629) polymorphism and gastric cancer 17. The variant genotype of *TNF*-α -308G>A was not associated with gastric atrophy (GA) in European studies 18,19 nor in meta-analysis 20, but has an impact on *H. pylori*-related gastroduodenal conditions like gastritis, ulcer or cancer 21. *TNF-*α -238G>A (rs361525) polymorphism was associated with the increased risk of gastric cancer in Chinese population, not in Caucasians 22,23 and was significantly associated with a high risk of gastritis in an African population 24.

Interleukin-6 (IL-6) is a multifunctional cytokine (endocrine and inflammatory mediator) and its polymorphism and expression seem to influence the susceptibility to various diseases, including gastric conditions related to *H. pylori* infection 25. Meta-analysis questioning the influence of *IL-6* promoter polymorphism did not reveal increased risk for gastric cancer 26. A study from Brasilia supported that the G allele frequency of *IL-6* -174C>G (rs1800795) was higher in patients with gastric cancer than in patients with chronic gastritis 27, while others did not support any influence on gastric conditions related with *H. pylori* infection (gastritis, ulcer, adenocarcinoma) 28.

The current concept supports that gastric atrophy can be a result of chronic *H. pylori* infection or of autoimmune gastritis; in the latter, the sensitized T cells and autoantibodies are the key factors of the pathological process 29. *H. pylori* infection and autoimmune atrophic gastritis face overlapping biological characteristics and the germ itself may accelerate progression toward atrophy in individuals with a particular genetic background 36.

To the best of our knowledge there are no published studies investigating the importance of cytokine gene variants with a role in the inflammatory response of gastric mucosa on the extension of AGIM with negative biopsies for active *H. pylori* related gastritis. Based on all the observations cited above, the present study focused on assessing the associations of the *IFN‐γ, TGF‐β1*, *TNF-α*, and *IL‐6* gene polymorphisms with a possible role in clinical course of gastric inflammatory response, with the histologic extent of AGIM in patients without active inflammatory cell infiltrate on histology (mononuclear and neutrophilic). We questioned the possible influence of inflammatory cytokine polymorphism on localization or extension of premalignant gastric lesions leading to different susceptibility for gastric cancer, irrespective of triggered mucosal aggression. We present the following article in accordance with the STROBE reporting checklist.

## Patients and Methods

### Ethical consideration

The Ethical Committee of Targu Mures County Emergency Clinical Hospital (10846/15.04.2019) and of George Emil Palade University of Medicine, Pharmacy, Science and Technology of Targu Mures, Romania (282/19.07.2019) approved the study.

### Study sample

We conducted a single-center observational study in 224 adult consecutive patients, in whom an upper digestive endoscopy was performed in Medical Clinic II -Targu Mures County Emergency Clinical Hospital. A structured direct interview was applied after the informed consent was obtained. We questioned smoking and alcohol consumption, present symptoms (pain and/or heartburns and/or nausea and/or regurgitation), and some non-recorded data of the past medical history. The medical records of subjects were checked for previous symptoms, diagnosis, investigations or treatments for peptic ulcer disease and/or *H. pylori* eradication therapies, as well as for concomitant diseases (hypertension, cardiac, respiratory, kidney or liver diseases, stroke, diabetes mellitus, atherosclerosis, dyslipidemia, osteoarticular diseases, other chronic medical conditions) or treatments with potential gastric effect (protective or aggressive).

Patients drinking at least 10 Units (1 Unit=10 ml of pure alcohol) weekly were considered drinkers. Subjects reporting consumption of less than 10 Units of pure alcohol per week were considered non-drinkers. If the patients used to smoke 5 or more cigarettes/d, including recent quitters (last 5 years), they were considered as smokers. Non-steroidal non-aspirin anti-inflammatory drugs (commonly known as NSAIDs) consumption was considered if the patients took regular daily doses of over-the-counter drugs or based on medical prescription, for more than 1 mo. We recorded the use of antiplatelet dose of aspirin (75-125 mg/d) or clopidogrel 75 mg/d for more than 1 mo. Patients were considered on acenocumarolum (ACO) therapy if they used regular doses for a therapeutic international normalized ratio for at least 2 week before endoscopy. Patients were considered exposed to proton pump inhibitors (PPI) (omeprazole, pantoprazole, esomeprazole) if they used regular doses within the last month, irrespective of the type of administration (continuous or on-demand).

Exclusion criteria were: 1. Incomplete set of histological or clinical data; 2. Active *H. pylori* related gastritis on histology using immunohistochemistry study; 3. Previous gastric surgery; 4. Active bleeding during endoscopy requiring hemostatic therapy; 5. Advanced or end-stage digestive disease (cirrhosis, esophageal varices); 6. Dysplasia or gastric cancer.

### Pathology

At least four biopsies (two from the antrum and two from the gastric body, both from the lesser and the greater curvature) were routinely analyzed. The cases with absence of *H. pylori* infection in all biopsies on microscopy after staining tissues with hematoxylin-eosin, periodic acid Schiff-alcian blue and Giemsa were considered negative. If the germ was present in at least one site, the case was considered *H. pylori-*positive and it was excluded. If *H. pylori* infection was suspicioned (abundant inflammatory cells, extensive intestinal metaplasia), an immunohistochemistry study was performed, especially in patients on PPI therapy and the case was also excluded if infection was confirmed. The Updated Sydney System was used to assess the degree of mucosal chronic inflammation and activity, *H. pylori* infection, glandular atrophy, and intestinal metaplasia. The lack of biopsies from incisura in some patients did not allow us to use de OLGA/OLGIM system to quantify the severity of premalignant lesions. Moreover, we did not intend to study the association of the SNPs, clinical and endoscopic variables with the severity of premalignant lesions, but only with their presence or absence.

In this study, we focused on the presence of AGIM in any part of the stomach. Antrum-limited AGIM was defined as the presence of AGIM in the antrum, with a healthy stomach corpus. Corpus-limited AGIM was defined as the presence of AGIM in the corpus, with a healthy antrum. Extended AGIM was defined as the simultaneous histological presence of AGIM in the antrum and corpus of the stomach. In patients with corpus limited changes, enterocromaffin-like cell hyperplasia and suspicion of autoimmune gastritis, further studies were performed to confirm the diagnosis (anti parietal cell antibody and anti-intrinsic factor, B12 vitamin and folate serum level). Patients with other abnormal histological changes were excluded.

### Genetic study

Rapid extraction of genomic DNA from whole blood samples stored in EDTA tubes was performed by using PureLink Genomic DNA Mini Kits (ThermoFisher Scientific, Waltham, MA, United States). For *Taq*Man SNP genotyping, we used *Taq*Man Fast Advanced Master Mix and the assay for *TNF-α* rs361525 and the 7500 Fast Dx Real*‐*time PCR System (ThermoFisher Scientific). *TGF‐β1* rs1800470, *TNF-α* rs1800629, *IFN‐γ* rs2430561, and *IL‐6* rs1800795 SNPs were evaluated by using the previously described amplification*-*refractory mutation system (commonly known as the ARMS‐PCR) technique 31.

### Statistical analysis

Statistical analysis was performed using the R software (version 3.6.1). Distribution of observed and expected genotypes of studied gene polymorphisms was tested for consistency with Hardy-Weinberg equilibrium and linkage equilibrium using the SNPassoc R package 32. Demographic variables, such as age, were described by mean and standard deviation, while other clinical categorical factors were shown using absolute frequencies and percentages. Comparisons between demographic and clinical factors among patients with different extensions of AGIM were performed using the Student's *t*, ANOVA, chi-square or Fisher's exact test.

The differences in genotype frequencies of *IFN‐γ*, *TGF‐β1, TNF‐α,* and *IL‐6* gene polymorphisms among patients with different extensions of AGIM were tested using chi-square or Fisher's exact test. In the case of a significant result (*P*-value < 0.05), in order to identify the pattern of differences, we also performed the pairwise comparisons using chi-square or Fisher's exact test, and then the *P*-values were adjusted using the Benjamini-Hochberg method 33.

Binomial and multinomial logistic regression analysis with adjustment for age, gender, and current smoking were performed to estimate the association between variant genotype of *IFN‐γ, TGF‐β1, TNF‐α,* and *IL‐6* gene polymorphisms and extension of the presence of AGIM. The association between each of the studied gene polymorphisms was expressed in terms of the odds ratio (OR) and the corresponding 95% confidence interval (CI). The estimated ORs were obtained using the VGAM R package 34. The regression results were considered statistically significant if 95%CI for OR did not contain unity or if two-tailed *P*-values obtained from Wald *z*-tests were lower than the significance level of 0.05.

## Results

### Study sample

Among the 224 consecutive patients included in the study, the frequency of AGIM was 73 (32.6%) cases. The histopathological investigation revealed that the extension of AGIM was the following: 37 (50.7%) patients had antrum-limited AGIM; 21 (28.8%) patients had corpus-limited AGIM; and 15 (20.5%) patients had extended AGIM, involving both antrum and corpus.

The main demographic and clinical variables are presented in Table 1. The mean age of studied patients was 62.3 ± 12.7 years, with range of age varying between a minimum of 20 years to a maximum of 85 years (significant differences found when the group with AGIM was compared to patients with no AGIM; Student's *t*-test, *P* = 0.004). We noticed that mean age of patients with AGIM was higher than of patients with no AGIM (65.52 ± 10.47 *vs* 60.79 ± 13.37 years). The distribution of gender was similar between the groups with and without AGIM (chi-square test, *P* = 0.776).

Although we observed that among patients with different histologic extensions of AGIM, the frequency of gastrotoxic drugs consumption (aspirin, non-aspirin NSAIDs) was higher for patients with antrum-limited AGIM (*n* = 19; 51.4%) versus corpus-limited AGIM (*n* = 8; 38.1%), versus extended AGIM (*n* = 7; 46.7%), the observed differences were not statistically significant (chi-square test, *P* = 0.733). The frequency of gastro-protective (PPI) drugs was similar according to different histologic extensions of AGIM: 105 (69.5%) for no AGIM *vs* 27 (73.0%) for antrum-limited AGIM *vs* 13 (61.9%) for corpus-limited AGIM *vs* 10 (66.7%) for extended AGIM (chi-square test, *P* = 0.874).

There was a significant association between ACO consumption and localization of AGIM (Fisher's exact test, *P* = 0.042), with the pairwise comparisons showing that the frequency of ACO consumption was higher among patients with corpus-limited AGIM than those with no AGIM (Fisher's exact test, *P* = 0.007, adjusted *P* = 0.041).

There were no significant differences regarding the frequency distribution in any of digestive symptoms for patients with antrum-limited AGIM, corpus-limited and extended AGIM (chi-square test, *P* = 0.168 for abdominal pain, *P* = 0.125 for pyrosis; Fisher's exact test, *P* = 0.624 for nausea and *P* = 0.895 for regurgitation; chi-square test, *P* = 0.230 for bloating).

There was a significant association between smoking and distribution pattern of AGIM (Fisher's exact test, *P* = 0.026), the post-hoc analysis identifying significant differences between patients with extended AGIM and those with antrum-limited AGIM (*P* = 0.015, adjusted *P* = 0.091) and those with corpus-limited AGIM (Fisher's exact test, *P* = 0.032, adjusted *P* = 0.097).

### Hardy-Weinberg equilibrium for IFN-γ, TGF-β1, TNF-α and IL-6 gene polymorphisms

The studied SNPs were tested for the condition of Hardy-Weinberg equilibrium. The results showed that genotype distribution of observed and expected frequency of each SNP did not differ significantly in patients with and without AGIM, all the studied SNPs (excepting *TNF-α* -238G>A, for which we found only two genotypes) being in agreement with Hardy-Weinberg equilibrium expectation; for the AGIM group and for patients without AGIM: *IFN-γ* +874T>A: *P* = 0.1543 and *P* = 0.6204; *TGF-β1* 869T>C: *P* = 0.8533 and *P* = 1.000; *TNF-α* -308G>A: *P* = 0.4437 and *P* = 0.1332; and *IL-6 -*174C>G: *P* = 1.0000 and *P* = 0.4968). We also tested linkage disequilibrium between *TNF-α* -308G>A and -238G>A SNPs and found no significant deviation from linkage disequilibrium (*P* = 0.801, D' = 0.047, *r*^2^ = 0.012).

### Association of TGF-β1, TNF-α, IFN-γ, and IL-6 gene polymorphisms with histologic extensions of AGIM

Table 2 summarizes the frequencies of studied SNPs' genotypes in relation to the histologic extension of AGIM. There was no significant difference regarding variant genotype and wild-type genotype frequencies among patients with different localization of AGIM, except for *TGF-β1* +869T>C gene polymorphism (chi-square test, *P* = 0.031). In addition, we noticed that the variant genotype *TGF-β1* +869T>C gene polymorphism occurred less frequently among patients with corpus-limited AGIM (*n* = 7, 33.3%) and extended AGIM (*n* = 5, 33.3%) than those with antrum-limited AGIM (*n* = 25, 67.6%).

As described in Table 3, the dominant inheritance models showed a significant association only for *TGF-β1* +869T>C gene polymorphism with decreased risk of corpus-affected AGIM (adjusted OR= 0.42, 95%CI: 0.19- 0.93, *P* = 0.032).

## Discussion

Our study investigated the environmental factors and their effects, as well as the *IFN‐γ, TGF‐β1*, *TNF‐α* and *IL‐6* gene polymorphisms of cytokines involved in the immune response, on patients with different extent of AGIM without active *H. pylori* infection on histology.

Identification of cytokines' functions and their cellular actions has increased during the last years, and our understanding of the link between inflammation and gastric carcinogenesis has advanced greatly. Even though gastritis associated with *H. pylori* infection mainly involving the antrum is distinct from atrophic autoimmune gastritis (implicating the gastric corpus because the inflammatory process affects the parietal gastric cells), the correlation between the two is still controversial 30. A recent meta-analysis supported that autoimmune mechanisms might exacerbate *H. pylori* gastritis in the absence of classical biological features of autoimmune gastritis 35. We did not intend in our study to clarify the etiology of gastric atrophy or metaplasia, but we started the research in order to investigate possible inflammatory gene polymorphisms that may modulate the increasing cases of *H. pylori*-negative biopsies in dyspeptic or anemic patients undergoing endoscopy, probably due to unintentional eradication or spontaneous disappearance of the germ in “old” atrophic gastritis 36. The corpus-limited lesions group include both patients with confirmed autoimmune gastritis or not.

The frequency of AGIM in the studied sample (32.6%) was three-times higher than the prevalence of intestinal metaplasia reported by Huang *et al.* 37 in a recent retrospective large American study of 17,710 biopsies, and also higher than that reported in European studies (one in four biopsies of patients undergoing gastroscopy) 38. Both genetic and environmental factors are accepted to play a role in this discrepancy, including the prevalence of *H. pylori* infection 39.

There were not significant differences regarding the symptoms or history of ulcer in patients with or without AGIM, nor in patients with different extent of histologic lesions. NSAID or aspirin consumption were not different in the studied groups, while ACO therapy was more frequent in patients with corpus-limited AGIM than in those with no AGIM. As it was reported, the use of ACO was frequently associated with the extension of preneoplastic lesions 40. Vitamin B12 and folic acid deficiency occurring in corporeal autoimmune gastritis are associated with variable increased homocysteine levels 41, which seem to increase the risk for thrombotic events 42. Even though the association remains controversial 43, the link between atrophic gastritis and thrombotic risk should be further investigated. Regarding the relation between smoking and histologic extent of AGIM, our findings revealed that smoking was associated with AGIM, as in other similar studies of dyspeptic patients 44, or with endoscopic lesions. The underlying mechanisms seem to be related to the influence on mucosal cell death, proliferation, decreased blood flow, or modulation of the immune responses in gastric mucosa 45.

Our data showed that* IFN-γ* +874T>A, *TNF-α* -308G>A and -238G>A, *IL-6 -*174C>G polymorphisms were not associated with the extent of AGIM in patients without active *H. pylori* infection immunohistochemically assessed. Similar results were observed in gastric cancer 12, 22, 23,26 and in gastric atrophy in Europeans 18,19, 20 but no data are available for patients without active *H. pylori* infection.

From among all the polymorphisms studied, only the *TGF-β1* +869T>C SNP was associated with localization of premalignant gastric lesions in patients without *H. pylori* infection. The multifunctional TGF-β1 proteins were proved to control cell growth, proliferation, differentiation and apoptosis and their role on carcinogenesis was extensively studied 11. In our study, the variant genotype *TGF-β1* +869T>C was associated with a protective effect against corporeal localization of AGIM. The findings suggest the possible effect of *TGF-β1* +869T>C, rs1800470 polymorphisms on modulation of the host immune response in gastric corpus mucosa. It is accepted today that *H. pylori* infection might mediate aggression against the proton pump, leading to corporeal atrophic mucosal changes similar to those of primary gastric autoimmunity, disappearing from altered mucosa 1. This overlapped pathogenic mechanism of gastric corpus atrophy might be influenced by the *TGF-β1* +869T>C SNP, modulating the cytokine activity that plays roles in gastric inflammation via regulatory mechanisms.

Our study is the first one questioning the role of cytokine polymorphisms in premalignant gastric lesions in patients with unintentional or spontaneous disappearance of *H. pylori* infection in gastric biopsies or with autoimmune gastritis, as recent studies underline the important roles for cytokines in regulating corporeal atrophy, hyperplasia, different types of metaplasia, and gastric carcinogenesis 46,47 Our study opens the possible direction of research for developing alternative biologic markers for assessment of risk for premalignant gastric lesions and associations with other medical conditions.

One limitation of the study is the lack of data regarding plasma cytokines* (IFN‐γ, TGF‐β1, TNF-α,* and *IL‐6*). The second limitation of our study was no separate estimation of the risk for both corpus-limited and extended AGIM, due to reduced numbers of cases with variant genotypes of the studied SNPs. Although the *TNF-α*-238G>A polymorphism TGF-β1 +869T>C gene polymorphism was significantly associated with decreased risk of corpus-affected AGIM, the results should be regarded with caution due to the small frequencies of variant genotype and the clinical significance should be retested on a larger sample. The final regression model does not include the *TNF-α-*238G>A polymorphism due to a lack of cases with the variant genotype and extension of AGIM into the gastric corpus and it was the third limitation of the present study. Further studies should investigate the effect of *TGF-β1*+869T>C, rs1800470 polymorphisms on chronic gastritis occurrence in a specific population. The fourth limitation was the small sample size, that did not allow for the development of a multivariable logistic model to study the clinical predictors together with *IFN‐γ, TGF‐β1, TNF-α,* and *IL‐6* gene for different localization or extension of AGIM. The small sample size of each group based on AGIM localization was also related to the wide confidence intervals of estimated parameters (cOR, aOR) so further studies with larger sample sizes should be made to get a greater precision of findings and to investigate the ability of multivariable clinical and genetic model to predict the extent of AGIM, as well as the importance of the past or present *H. pylori* infection.

## Conclusions

In patients without active *H. pylori* gastritis in biopsy samples, the *TGF-β1* +869T>C gene polymorphism was associated with a decreased risk for corporeal localization of AGIM. The dominant inheritance models revealed no significant association for *IFN-γ* +874T>A, *TNF‐α*-308G>A and *IL-6 -*174C>G gene polymorphism with the risk of localization of AGIM. Higher consumption of ACO was observed in patients with corpus-limited precancerous lesions, while symptoms were not associated with localization of premalignant lesions. In patients without active *H. pylori* infection smoking was associated with extended AGIM.

## Figures and Tables

**Table 1:** Demographic and clinical features in patients with different extensions of atrophic gastritis and/or intestinal metaplasia

Variable	AGIM in any stomach site, *n* = 73 (32.6%)	Absence of AGIM, *n* = 151 (67.4%)	Antrum-limited AGIM, *n*_1_ = 37 (16.5%)	Corpus-limited AGIM, *n*_2_ = 21 (9.4%)	Extended AGIM, *n*_3_ = 15 (6.7%)
Age in years, mean ± SD	65.52 ± 10.47^1^	60.79 ± 13.37	64.51 ± 10.18	66.67 ± 10.62	66.40 ± 11.42
Gender, male	34 (46.6)	67 (44.4)	15 (40.5)	11 (52.4)	8 (53.3)
Peptic ulcer history	47 (64.4)	95 (62.9)	23 (62.2)	14 (66.74)	10 (66.7)
Non-aspirin NSAIDs use	9 (12.3)	15 (9.9)	6 (16.2)	1 (4.8)	2 (13.3)
Aspirin use	29 (39.7)	63 (41.7)	17 (45.9)	7 (33.3)	5 (33.3)
ACO use	20 (27.4)^*^	24 (15.9)	8 (21.6)	9 (42.9)^**^	3 (20.0)
Clopidogrel use	13 (17.8)	19 (12.6)	12 (32.4)	0 (0.0)	1 (6.7)
PPI use	50 (68.5)	105 (69.5)	27 (73.0)	13 (61.9)	10 (66.7)
**Digestive symptoms**					
Abdominal pain	30 (41.1)	82 (54.3)	17 (45.9)	9 (42.9)	4 (26.7)
Pyrosis	15 (20.8)	41 (27.2)	11 (30.6)	1 (4.8)	3 (20.0)
Nausea/vomiting	13 (17.8)	22 (14.6)	6 (16.2)	3 (14.3)	4 (26.7)
Bloating	21 (28.8)	34 (22.7)	14 (37.8)	4 (19.0)	3 (20.0)
Regurgitation	3 (4.1)	8 (5.3)	1 (2.7)	1 (4.8)	1 (6.7)
Comorbidities**^†^**	72 (98.6)	144 (95.4)	37 (100.0)	21 (100.0)	14 (93.3)
Current smoking	9 (12.3)^*^	5 (3.3)	4 (10.8)^***^	3 (14.3)^****^	2 (13.3)
Alcohol consumption, > 10 u/wk	7 (9.6)	11 (7.3)	2 (5.4)	2 (9.5)	3 (20.0)

Data are presented as *n* (%), unless indicated otherwise. ^*^P < 0.05 obtained from Student's *t*-test/Fisher's exact test, applied to compare the AGIM in any stomach site and no AGIM groups; ^**^P < 0.05 obtained from Fisher's exact test, applied to compare the corpus-limited AGIM and no AGIM groups; ^†^ presence of at least one comorbidity as described in Methods; ^***^P < 0.05 obtained from Fisher's exact test, applied to compare the antrum-limited AGIM and extended AGIM; ^****^P < 0.05 obtained from Fisher's exact test, applied to compare the corpus-limited AGIM and extended AGIM groups. ACO: Acenocumarolum; AGIM: Atrophic gastritis and/or intestinal metaplasia; NSAID: Non-steroidal non-aspirin anti-inflammatory drug; SD: Standard deviation.

**Table 2:** Genotype frequencies of *IFN*‐γ, *TGF*‐β1,* TNF*‐α, and *IL‐6* gene polymorphisms in patients with different histologic extensions of AGIM

Gene polymorphism, n (%)	AGIM in any site [n=73 (32.6)]	Absence of AGIM [n=151 (67.4)]	p-value*	Antrum-limited AGIM [n_1_=37 (16.5)]	Corpus-limited AGIM [n_2_=21 (9.4)]	Extended AGIM [n_3_=15 (6.7)]	p-value**
***IFN*‐γ +874T>A**							
TT	11 (15.1)	31 (20.5)	0.244	5 (13.5)	4 (19.0)	2 (13.3)	0.440
AT	43 (58.9)	71 (47.0)		19 (51.4)	13 (61.9)	11 (73.3)	
AA	19 (26.0)	49 (32.5)		13 (35.1)	4 (19.0)	2 (13.3)	
AT+AA	62 (84.9)	120 (79.5)	0.326	32 (86.5)	17 (81.0)	13 (86.7)	0.814
***TGF*-β1 +869T>C**							
TT	36 (49.3)	69 (45.7)	0.747	12 (32.4)	14 (66.7)	10 (66.7)	0.123
CT	31 (42.5)	65 (43.0)		21 (56.8)	5 (23.8)	5 (73.3)	
CC	6 (8.2)	17 (11.3)		4 (10.8)	2 (9.5)	0 (0.0)	
CT+CC	37 (50.7)	82 (54.3)	0.611	25 (67.6)	7 (33.3)	5 (33.3)	0.031
***TNF-α* -308G>A**							
GG	46 (63.0)	92 (60.9)	0.953	24 (64.9)	15 (71.4)	7 (46.7)	0.719
AG	26 (35.6)	56 (37.1)		12 (32.4)	6 (28.6)	8 (53.3)	
AA	1 (1.4)	3 (2.0)		1 (2.7)	0 (0.0)	0 (0.0)	
AG+AA	27 (37.0)	59 (39.1)	0.763	13 (35.1)	6 (28.6)	8 (53.3)	0.481
***TNF-α* -238G>A**							
GG	71 (97.3)	146 (96.7)	1.00	35 (94.6)	21 (100.0)	15 (100.0)	0.799
AG	2 (2.7)	5 (3.3)		2 (5.4)	0 (0.0)	0 (0.0)	
***IL-6 -*174C>G**							
GG	23 (31.5)	58 (38.4)	0.598	11 (29.7)	8 (38.1)	4 (26.7)	0.555
CG	37 (50.7)	68 (45.0)		20 (54.1)	11 (52.4)	6 (40.0)	
GG	13 (17.8)	25(16.6)		6 (16.2)	2 (9.5)	5 (33.3)	
CG+CC	50 (68.5)	93(61.6)	0.313	26 (70.3)	13 (61.9)	11 (73.3)	0.656

*P-values resulted from the comparison of genotype distributions in patients with and without AGIM by chi-square or Fisher's exact tests; **P-values resulted from the comparison of genotype distributions according to the distribution pattern of AGIM by chi-square or Fisher's exact tests. Statistical significance was reached if *P*-value < 0.05. AGIM: Atrophic gastritis and/or intestinal metaplasia.

**Table 3:** Associations between *IFN‐*γ,* TGF*‐β1, *TNF*‐α and *IL‐6* gene polymorphisms and extensions of atrophic gastritis and/or intestinal metaplasia

Dominant inheritance models of studied SNPs	AGIM in any site, *n* = 73		Antrum-limited AGIM, *n*_1_ = 37		Corpus-affected (limited+extended) AGIM, *n*_2_=36	
	cOR [95%CI]	aOR [95%CI]	cOR [95%CI]	aOR [95%CI]	cOR [95%CI]	aOR [95%CI]
***IFN*‐γ +874T>A**						
TT	Reference	Reference	Reference	Reference	Reference	Reference
AT+AA	1.46 [0.70, 3.21]	1.34 [0.64, 3.00]	1.65 [0.60, 4.59]	1.57 [0.56, 4.40]	0.97 [0.49, 3.38]	0.97 [0.43, 3.09]
***TGF*-β1 +869T>C**						
TT	Reference	Reference	Reference	Reference	Reference	Reference
CT+CC	0.86 [0.49, 1.51]	0.91 [0.51, 1.62]	1.75 [0.82, 3.75]	1.85 [0.86, 4.01]	0.42 [0.20, 0.90]^*^	0.42 [0.19, 0.93]^*^
***TNF-α* -308G>A**						
GG	Reference	Reference	Reference	Reference	Reference	Reference
AG+AA	0.92 [0.51, 1.62]	1.03 [0.56, 1.87]	0.84 [0.40, 1.79]	0.94 [0.44, 2.01]	0.99 [0.47, 2.09]	1.14 [0.53, 2.47]
***IL-6 -*174C>G**						
GG	Reference	Reference	Reference	Reference	Reference	Reference
CG+CC	1.36 [0.75, 2.48]	1.37 [0.75, 2.55]	1.47 [0.68, 3.21]	1.49 [0.68, 3.27]	1.25 [0.58, 2.68]	1.26 [0.57, 2.79]

*P*-values obtained from Wald *z*-tests of multinomial logistic regression. aOR: Adjusted odds ratio for gender, age older than 60 years, and current smoking; CI: Confidence interval; cOR: Crude odds ratio; AGIM: Atrophic gastritis and/or intestinal metaplasia; SNPs: Single nucleotide polymorphisms.
